# The role of vitamin D in the onset and progression of diabetic retinopathy

**DOI:** 10.22336/rjo.2022.42

**Published:** 2022

**Authors:** Geanina Totolici, Carmen Tiutiuca, Sanda Jurja, Dana Tutunaru, Andra-Mădălina Pătrașcu

**Affiliations:** * “Dunărea de Jos” University of Galaţi, Faculty of Medicine and Pharmacy, Galaţi, Romania; ** “Sf. Apostol Andrei” County Clinical Emergency Hospital, Galați, Romania; *** “Ovidius” University of Constanţa, Faculty of Medicine and Pharmacy, Constanţa, Romania; **** Municipal Emergency Hospital, Pașcani, Romania

**Keywords:** vitamin D, diabetes mellitus, diabetic retinopathy, inflammatory cells

## Abstract

Having a classical role in the regulation of calcium homeostasis in skeletal system, vitamin D has also been recognized as being involved in the activity of the immune system, as well as in the pathology of the visual analyzer.

Thus, regarding the function of vitamin D in the eye, this is supported by the identification of vitamin D receptors (VDR) in several structures of the eyeball, such as corneal epithelial and endothelial cells, ciliary body and retinal cells.

One of the ophthalmological pathologies in which vitamin D plays an important role is diabetic retinopathy, both through its effects on the immune system (reduction of the pro-inflammatory cytokines - IL-1, IL-6, IL-012, TNF alpha, and stimulation of anti-inflammatory cytokines IL-10), as well as by reducing the level of vascular endothelial growth factor (VEGF) and thus inhibiting retinal neovascularization.

Vitamin D demonstrates a protective role on the development and progression of diabetic retinopathy by reducing blood sugar, hypertension and atherosclerosis, but randomized studies are still needed to establish the direct causal relationship between the development of diabetic retinopathy and vitamin D levels.

## Introduction

The classic effect of vitamin D is to regulate calcium homeostasis in the bone system. However, in recent years, vitamin D has attracted the attention of the medical and scientific communities with its non-classical roles, such as: regulating hormone secretion or regulating cell differentiation. Through all these roles, vitamin D does not act as a vitamin in the strict sense, but rather acts as a prohormone [**1**,**2**]. 

There are two forms of vitamin D, namely D2 (ergocalciferol) and D3 (cholecalciferol) [**3**].

Vitamin D2 is formed by the action of ultraviolet radiation on ergosterol. Ergosterol sources come only from foods such as fatty fish (salmon, mackerel, sardines), cod liver oil and certain types of fungi [**3**,**4**].

Vitamin D3 is synthesized in the skin tissue by the action of ultraviolet B (UVB) radiation on 7-dehydrocholesterol (7-DHC). Physiologically, there are large amounts of 7-DHC in the subcutaneous tissue. Under the action of UVB, 7DHC is photolyzed and transformed into previtamin D3 [**5**,**6**].

The active form of vitamin D is 1,25-dihydroxy D3 (calcitriol) and is obtained by enzymatic conversion of D3 by 25-hydroxylation and 1-α-hydroxylation [**7**].

25-hydroxylation takes place in the liver under the action of enzymes, and 25 hydroxy vitamin D (25 (OH) D) is formed as a result of this process. Serum dosage of this metabolite (25 (OH) D) reflects the nutritional status of vitamin D [**7**].

Although 1-α-hydroxylase is present in different types of cells (bone cells, immune system cells, placental cells), 1-α-hydroxylation occurs in the renal proximal tubule, where there is the highest concentration of this enzyme [**8**,**9**].

To avoid hypervitaminosis by excessive sun exposure, both 25 (OH) D and 1,25 (OH)2D, undergo the enzymatic process of 24-hydroxylation as a result of which they become inactive forms [**8**,**9**].

Vitamin D deficiency (VD) is a global problem, some studies reporting its presence in 82.5% of subjects, with an increased frequency even in countries with year-round sunlight. VD deficiency is given by serum levels of 25 (OH) D below 20 ng/ ml [**10**,**11**].

The main risk factors for vitamin D deficiency are:

- age over 70 years;

- use of sunscreen;

- pigmented skin;

- Northern latitudes;

- obesity;

- not being exposed to the sun (work at home);

- medication: antiepileptics, antiretrovirals;

- liver or kidney diseases;

- malabsorption syndrome [**12**].

## The immunological role of vitamin D

The first links between vitamin D and the immune system emerged from reports of treatment of patients infected with Mycoplasma Tuberculosis. The benefits of exposing patients to UVB and administrating cod liver oil have been observed [**13**].

Numerous studies performed both in vitro and in vivo have shown that the enzymes required for the activation of vitamin D and receptors of vitamin D (VDR) are present in the cells belonging to the immune system, such as T lymphocytes, B lymphocytes, natural killer cells (NK), monocytes and neutrophils [**14**].

Vitamin D regulates the activity of T lymphocytes, both directly through its endocrine and paracrine function and indirectly by modulating the stimulatory function of T lymphocytes on antigen presenting cells (APC) [**1**].

Because of its suppression of T lymphocyte proliferation, there is a decrease in the production of pro-inflammatory cytokines (IL-17, IL21) and an increase in anti-inflammatory cytokines (IL-10) [**1**,**15**].

The same effect of reduction of inflammatory cytokines (IL-1, IL-6, IL-12, and TNF) is obtained by the action of vitamin D on monocytes [**1**,**15**].

The active form of vitamin D induces apoptosis of B lymphocytes having a role in maintaining homeostasis and providing protection against autoimmune diseases based on their proliferation [**16**]. 

Vitamin D receptors also exist in neutrophils and NK cells, thus enhancing their function of defense against pathogens, by increasing cathelicidin and α and β defensin, enzymes that destroy the bacterial cell membrane [**15**,**17**].

Although more and more evidence show the link between vitamin D deficiency and the risk of developing or progressing certain autoimmune/ inflammatory diseases such as: multiple sclerosis, rheumatoid arthritis, inflammatory bowel disease, systemic lupus erythematosus (SLE), diabetes, atherosclerosis, the causality of this association remains to be established by randomized clinical trials [**1**,**18**].

## Vitamin D and ocular structures

The first correlations between vitamin D and the eyeball were established by Verstappen et al. in 1986, when they identified the presence of calbindin (a specific vitamin D transport protein) in the human retina [**19**]. 

Later, in 1995, Johnson et al. identified by immunohistochemical staining the presence of VDR in the corneal endothelium, ganglion cells and photoreceptor cells [**20**].

In 2014, Alsalem et al. determined the presence of hydroxylase (required for the activation of vitamin D) in corneal epithelial cells, cells of the ciliary body, the retina cells, thus showing that these cells are capable of conversion of vitamin D from the inactive to the active form [**21**,**22**].

The presence of VDR and hydroxylase in the eyeball cells suggests that vitamin D may have a favorable role in the prevention and evolution of various eye diseases such as:

• myopia;

• retinoblastoma;

• age-related macular degeneration;

• glaucoma;

• uveitis;

• dry eye syndrome;

• diabetic retinopathy [**21**]. 

## The role of vitamin D in the prevention of diabetic retinopathy

In recent years, diabetes mellitus (DM) has become an important public health problem due to its continuously increasing prevalence. Epidemiological studies estimate the number of patients with DM to increase from 463 million in 2019 to 700 million in 2045 [**23**].

Diabetic retinopathy (DR) is a frequent microvascular complication of DM and one of the main causes of blindness worldwide, especially among the active population aged between 20-60 years [**24**].

The main modifiable risk factors for the development of DR are: hyperglycemia, arterial hypertension, obesity, dyslipidemia. Non-modifiable risk factors are: genetic factors and DM duration [**6**]. In terms of duration, studies showed that after 20 years of DM, almost all patients with type I DM and over 60% of patients with type II DM have developed some form of diabetic retinopathy [**25**].

Thus, DR is a multifactorial condition that is etiopathogenically characterized by hyperglycemia, chronic inflammation, leukostasis, microvascular lesions associated with increased vascular permeability, local ischemia, angiogenesis and neurodegeneration [**6**]. 

Among the multitude of etiopathogenic factors involved in the occurrence of DR, in recent years the inflammatory and angiogenesis processes have captured the attention of scientific researchers, becoming the center of multiple specialized studies [**25**].

The chronic inflammation present in any stage of DR, causes the local and systemic increase of inflammatory molecules such as: cell adhesion molecules, cytokines, chemokines, vascular endothelial growth factor (VEGF). The increased level of these molecules leads to the activation of leukostasis, which causes capillary occlusion with retinal hypoxia and damage to the endothelial cells, the result being the breakdown of the blood-retinal barrier (BRB). The destruction of the blood-retinal barrier leads to the appearance of exudates, haemorrhages and retinal edema [**25**,**27**]. 

More and more studies show that through its non-traditional roles such as: cell differentiation, modulation of inflammation, regulation of innate and acquired immunity, vitamin D is involved in the pathogenic processes underlying the development of DM and implicitly RD [**26**].

Through its effects on the immune system, vitamin D is also involved in the pathophysiological process of the development of diabetic retinopathy.

In patients with type II diabetes, inflammatory cytokines such as TNF α, TNF β, IL-6 are increased. Because of its action on T lymphocytes, vitamin D decreases the production of pro-inflammatory cytokines and increases cytokines with anti-inflammatory effect [**28**].

Complications of diabetic retinopathy that threaten vision loss are macular edema and neovascularization (proliferative DR), both of which are caused by increased VEGF production. Recently, in vitro studies have shown that the active form of vitamin D, calcitriol, has an effect of inhibiting neovascularization by reducing VEGF levels [**29**,**30**].

Numerous studies classify RD as sight-threatening (STDR) and non-sight-threatening (NSTDR). The inclusion criteria in the STDR stage are: the presence of severe non-proliferative diabetic retinopathy, proliferative retinopathy and the presence of diabetic macular edema regardless of the DR stage. The criteria for the NSTDR stage are the presence of mild or moderate nonproliferative diabetic retinopathy [**31**].

Based on this classification, a systematic review and meta-analysis that included 12 specialized studies and 9057 patients with DM, reported that vitamin D deficiency below 20 ng/ ml significantly increases the risk of STDR, but it was not associated with NSTDR [**31**].

On long term, hyperglycemia causes the formation of reactive oxygen species (ROS). At the level of the retina, the increase in the production of ROS at the level of endothelial cells, in pericytes, in Muller cells, in photoreceptor cells, results in cells apoptosis, which ultimately causes a decrease in visual acuity [**26**,**32**].

On the one hand, vitamin D reduces the insulin resistance of the body, and on the other, reduces oxidative stress by preserving mitochondrial function and decreasing the activity of monoamine oxidases [**29**,**33**].

Through the effect of lowering insulin resistance, vitamin D ensures better glycemic control, thus providing protection against the onset and progression of DR [**34**].

In addition, Payne et al. showed that patients without diabetes have higher serum levels of 25 (OH) D than those with type II diabetes, especially compared to those with proliferative diabetic retinopathy [**28**,**35**].

Similar to Payne’s study, Alcubierre et al. showed that patients with DR have a lower level of 25 (OH) D than those without DR [**36**]. 

Although multiple studies support the protective effect of vitamin D on the development of DR, its role in the etiopathogenesis of DR is incompletely elucidated and continues to be a controversial topic of debate in the medical community.

## Conclusions

Vitamin D is a multifunctional hormone that, in addition to its endocrine function, acts in a para or autocrine manner. In addition to the classic role of homeostasis of calcium, vitamin D has an important role in the cells of the immune system. 

Both vitamin D receptors and the enzymes required to activate it are found in the eye, suggesting that vitamin D is involved in the appearance and evolution of various ocular diseases. 

Regarding diabetic retinopathy, observational studies have indicated that low serum 25 (OH) D levels are associated with an increased risk of DR.

Through its anti-inflammatory effects, inhibition of angiogenesis and involvement in the process of cellular apoptosis, vitamin D has a beneficial role on the evolution of diabetic retinopathy, but randomized studies are needed to establish the direct causal relationship.

**Conflict of interest statement**

The authors state no conflict of interest.

**Acknowledgements**

None.

**Sources of Funding**

None.

**Disclosures**

None.
